# Supplementary material for: Identification of photoperiod-induced specific miRNAs in the adrenal glands of Sunite sheep (Ovis aries)
Source: Front Vet Sci. 2022 Jul 22;9:888207. doi: 10.3389/fvets.2022.888207 (PMC9354845; doi:10.3389/fvets.2022.888207)
Supplement: Supplementary file 1 [file Data_Sheet_1.ZIP › Supplementary file/miRDeep2 file.zip!/miRDeep2 file/Novel_Structure/SP42A1/Novel_197.pdf]

Provisional ID	:	NC_019459.2_2768
Score total	:	2.6
Score for star read(s)	:	-1.3
Score for read counts	:	0
Score for mfe	:	2.3
Score for randfold	:	1.6
Score for cons. seed	:	
Total read count	:	173
Mature read count	:	173
Loop read count	:	0
Star read count	:	0

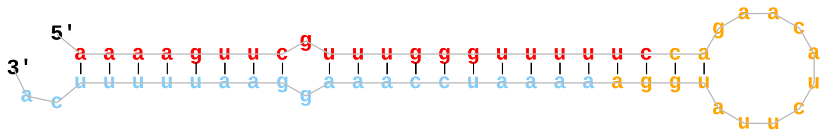

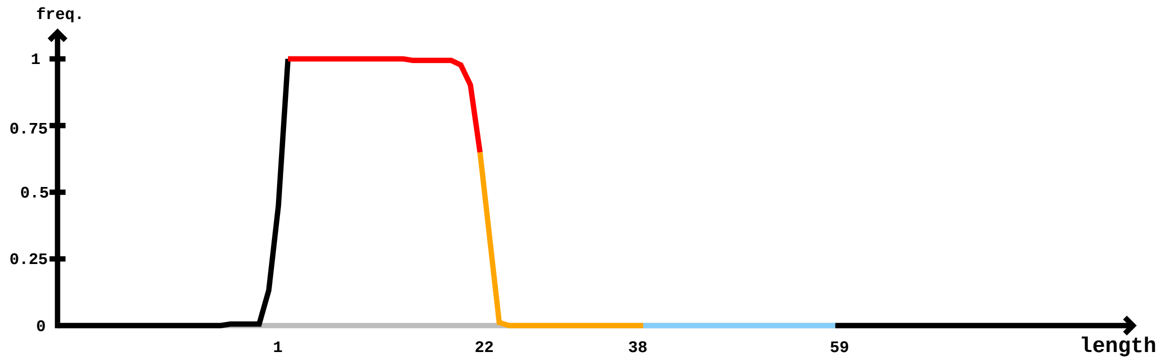

Mature **Star**

```
5'- ucaaauaauauugguuggcugaaaaguucguuuggguuuuuccagaacaucuuauggaaaaauccaaaggaauuuuucagccaacacaauauuaacuuucacagcuuug -3'   exp
    .....((((((((.((((((((((((((((.((((((((((((((..........)))))))))))))).)))))))))))))))).))))))))..............   reads   mm   sample
    ...............uggcugaaaaguucAuuug...........................................................................   1       1    seq
    ...................ugaaaaguucguuuggguuu......................................................................   1       0    seq
    ...................ugaaaaguucguuuggguuuuu....................................................................   21      0    seq
    ....................Aaaaaguucguuuggguuu......................................................................   2       1    seq
    ....................Aaaaaguucguuuggguuuu.....................................................................   12      1    seq
    ....................gaaaaguucguuuggguuuuu....................................................................   3       0    seq
    ....................Aaaaaguucguuuggguuuuuc...................................................................   38      1    seq
    .....................aaaaguucguuugAguuuu.....................................................................   1       1    seq
    .....................aaaaguucguuuggguuuuu....................................................................   20      0    seq
    .....................aaaaguucguuuggguuuuuc...................................................................   43      0    seq
    .....................aaaaguucguCuggguuuuuc...................................................................   1       1    seq
    .....................aaaaguucguuuggguuuuuA...................................................................   2       1    seq
    .....................aaaaguucguuuggguuuuuU...................................................................   5       1    seq
    .....................aaaaguucguuuggguuuuucA..................................................................   22      1    seq
    .....................aaaaguucguuuggguuuuucAa.................................................................   2       1    seq
```
